# Supplementary material for: Comprehensive human cell-type methylation atlas reveals origins of circulating cell-free DNA in health and disease
Source: Nat Commun. 2018 Nov 29;9:5068. doi: 10.1038/s41467-018-07466-6 (PMC6265251; doi:10.1038/s41467-018-07466-6)
Supplement: Supplementary file 1 — Supplementary Information [file 41467_2018_7466_MOESM1_ESM.pdf]

Supplementary Figures

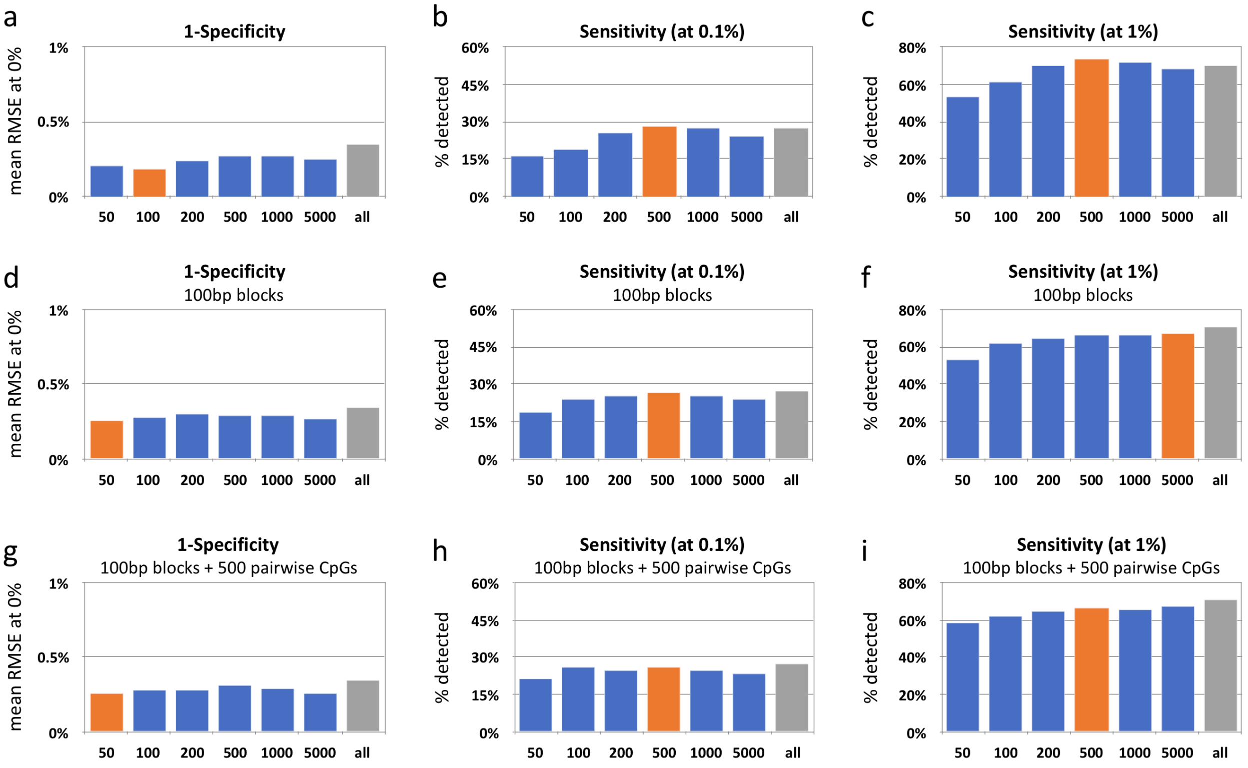

Supplementary Figure 1. Estimations of specificity (false positive rate) and sensitivity (detection rate) for various CpG selection strategies. (a) Specificity values, estimated as the mean error (RMSE) for simulations with 0% mixing (namely, pure leukocytes), for convolution with different numbers of selected CpGs per cell types. These include the top K differentially hypermethylated CpGs and top K hypomethylated CpGs per cell types, with K varying from 50 CpGs (50x2x25=2500 total), through 100, 200, 500, 1000, 5000, or all methylation array CpGs (right-most column). Note that deconvolution with all CpGs is less accurate (and less efficient) compared to models with fewer, selected, CpGs. **(b)** Sensitivity values, estimated as percent of in silico mixes (at 0.1%) correctly detected. Orange bar marks optimal selection (500 hyper + 500 hypomethylated CpGs per cell type, for total of 25,000 CpGs). **(c)** Same as (b), but at a 1% mixing-in level. **(d-f)** Same as (a-c), with deconvolution also based on all neighboring CpGs (up to 50bp away) from previously selected ones. The addition of neighboring CpGs allows for accurate deconvolution with few CpGs, e.g. 2x100 CpG blocks per cell type (total of 7,390 CpGs in 4,039 CpG "haplotype blocks"). **(g-i)** Specificity using previous CpGs with additional pairwise-specific 500 CpGs that are specifically selected to distinguish between similar cell types (e.g. different T cells, adipocytes vs. vascular endothelial cells, Bladder vs Prostate, etc), allowing for a further improvement in sensitivity with few CpGs.

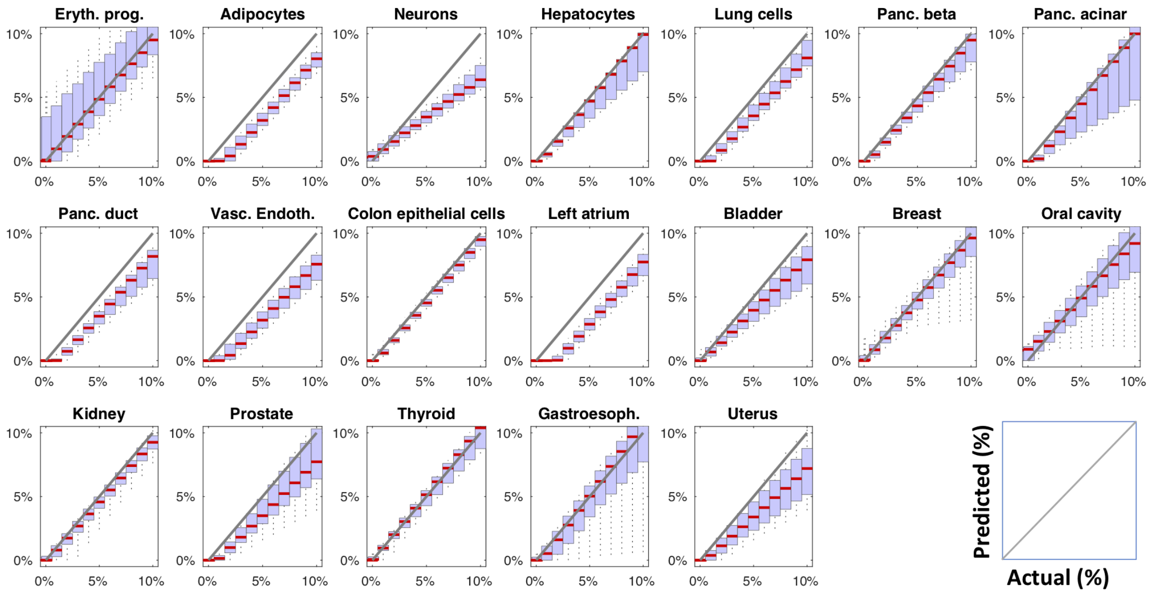

Supplementary Figure 2. Same as Fig 2, without feature selection (250,777 CpGs in total). Included are all CpGs, except for those with missing values or those with variance < 0.1% across the methylation atlas.

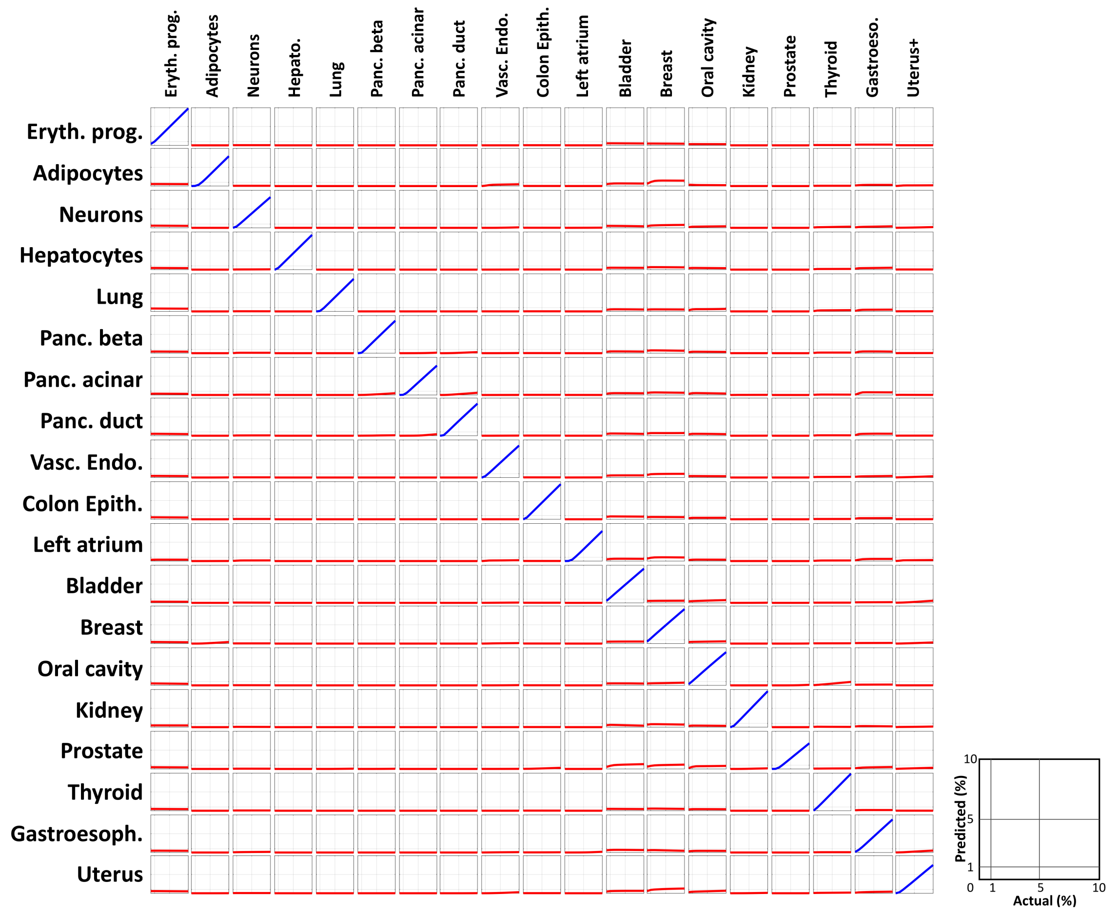

Supplementary Figure 3. Confusion matrix for deconvolution of plasma cfDNA methylomes. Each row corresponds to one cell type, in silico admixed with leukocytes at various mixing ratios from 0% to 10% (x-axis) and depicts the inferred proportion of the mixed (in blue) and all other cell types (in red). Most cell types are completely invariant of each other.

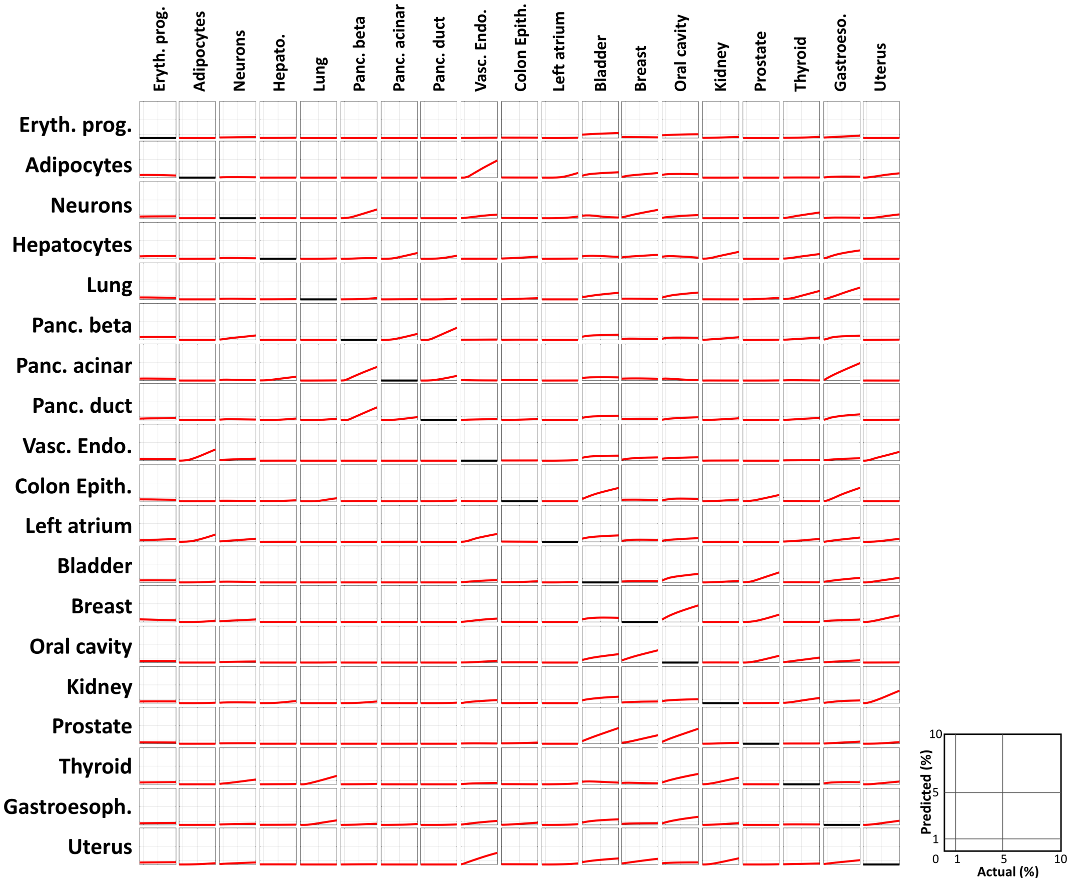

Supplementary Figure 4. Confusion matrix for deconvolution of plasma cfDNA methylomes. Unlike Supplementary Figure 3, here the admixed cell type was completely removed from the methylation atlas prior to deconvolution (black lines), resulting with some confusion between functionally or biologically related cell types (adipocytes and endothelial cells, pancreatic cells, etc).

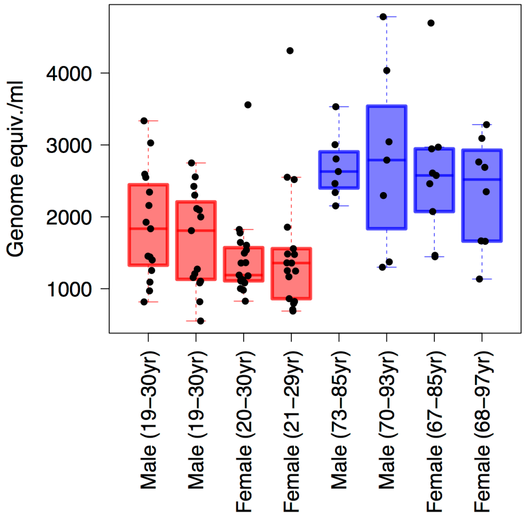

Supplementary Figure 5. cfDNA concentration of healthy individuals. Total concentration of cfDNA (in haploid genome equivalents/ml plasma) from all samples used to generate healthy pools are shown, grouped by the pool they were used in, as in Fig 4b. The cfDNA concentrations of the older individuals (blue boxplots) were significantly greater than those of the younger individuals (red boxplots) (p-value < 3.96e-7, Mann-Whitney test).

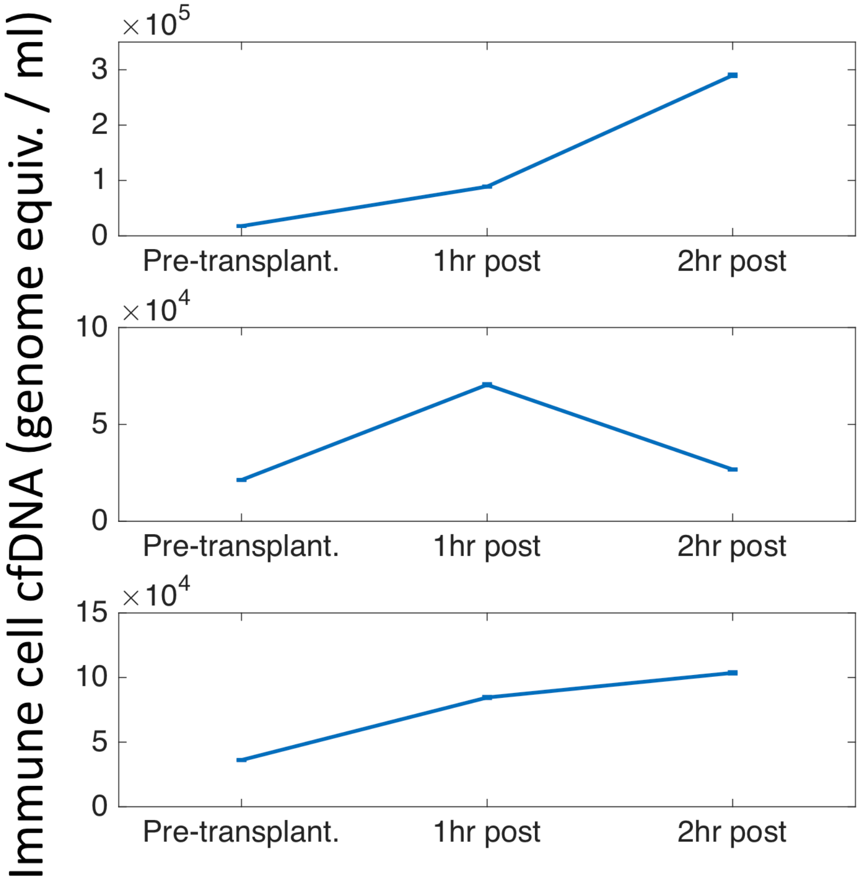

Supplementary Figure 6. Immune cell cfDNA in pancreatic islet transplantation. Same as Figure 5c, plotting the inferred amount of cfDNA (in haploid genome equivalents/ml plasma) from all immune cell types for three individuals prior to, 1 hour after, and 2 hours after islet transplantation. Error bars: SD, estimated using Bootstrapping.

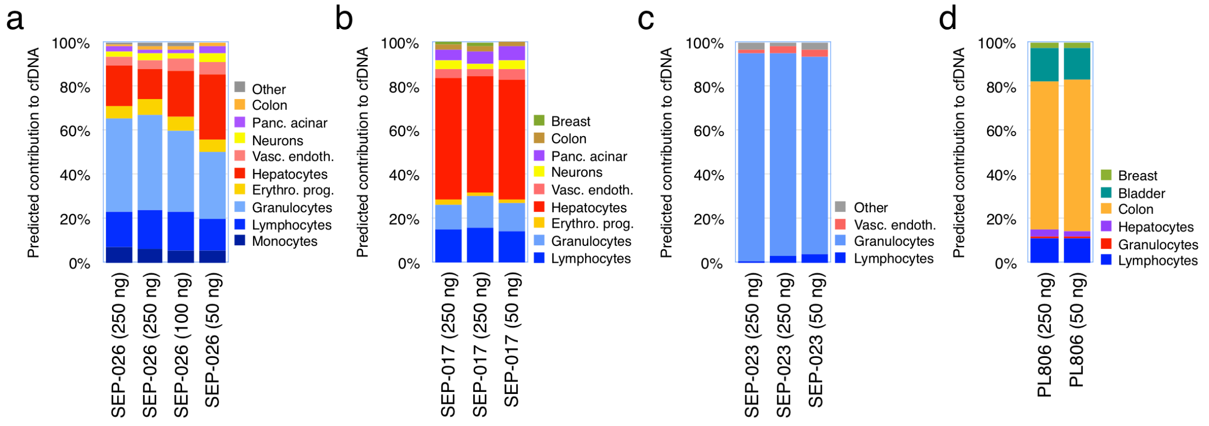

e

	SEP-026 (250 ng)	SEP-026 (250 ng)	SEP-026 (100 ng)	SEP-026 (50 ng)
SEP-026 (250 ng)		0.99	0.992	0.925
SEP-026 (250 ng)	1E-18		0.973	0.872
SEP-026 (100 ng)	1E-19	3E-14		0.956
SEP-026 (50 ng)	7E-10	2E-06	5E-12	

	SEP-017 (250 ng)	SEP-017 (250 ng)	SEP-017 (50 ng)
SEP-017 (250 ng)		0.998	0.999
SEP-017 (250 ng)	1E-24		0.996
SEP-017 (50 ng)	5E-31	1E-24	

	SEP-023 (250 ng)	SEP-023 (250 ng)	SEP-023 (50 ng)
SEP-023 (250 ng)		1	1
SEP-023 (250 ng)	1E-35		1
SEP-023 (50 ng)	2E-32	1E-37	

	PL806 (250 ng)	PL806 (50 ng)
PL806 (250 ng)		1
PL806 (50 ng)	1E-38	

Supplementary Figure 7. Reproducibility of deconvolution results. (a-d) Predicted distribution of cellular contributors shown for four samples using different amounts of DNA (50 ng, 100 ng or 250 ng). **(e)** Pearson correlation coefficients (in blue) and p-values (in yellow) shown for different pairs of analyzed plasma cfDNA methylomes (each set from the same individual).

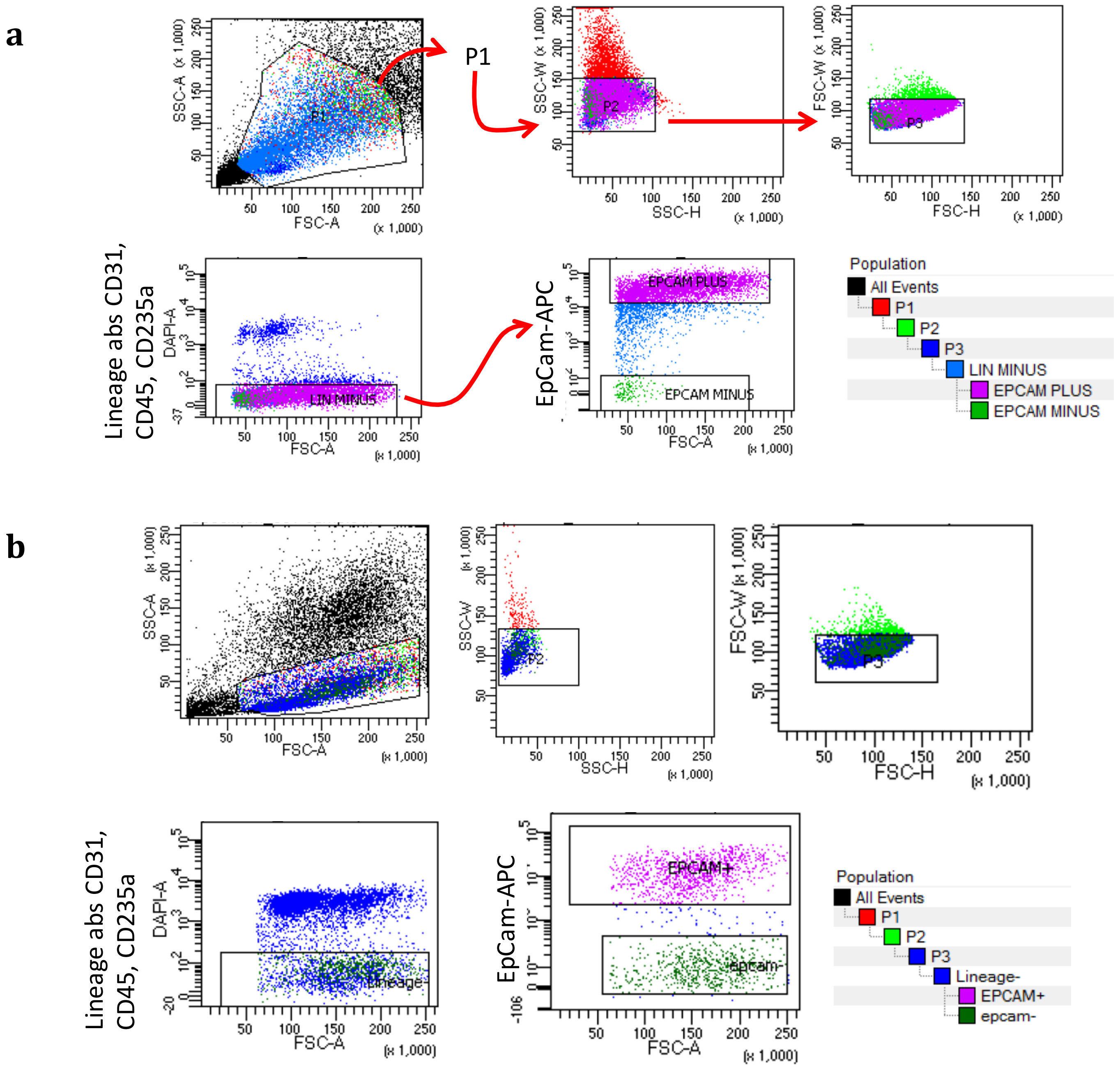

Supplementary Figure 8. Sorting and gating strategies for colon epithelial cells (a) and lung alveolar epithelial cells (b). We chose a P1 population according to size and granularity cell distribution, followed by gate P2 and then gate P3 to avoid doublets. From the P3 gate we chose the Lineage negative cells, and then selected the EpCam positive cells.

Legends for Supplementary Data file 1:

Supplementary Data File 1 contains nine individual tables, covering:
Table 1: Cell type-specific CpGs selected for deconvolution.
Table 2: Pairwise-differential CpGs selected for deconvolution.
Table 3: Ages of samples used in healthy pools.
Table 4: Inferred composition of healthy plasma cfDNA.
Table 5: Reference sample donor data.
Table 6: In vitro mixes.
Table 7: Cancer patient data.
Table 8: Healthy and cancer cfDNA mixes.
Table 9: Cancer of unknown primary site (CUP) data.
